# The influence of visible light exposure on cultured RGC-5 cells

**Published:** 2007-02-11

**Authors:** John P. M. Wood, Gerassimos Lascaratos, Anthony J. Bron, Neville N. Osborne

**Affiliations:** Nuffield Laboratory of Ophthalmology, University of Oxford, Oxford, United Kingdom

## Abstract

**Purpose:**

To determine the effects of visible light on normal or metabolically compromised cultured rat RGC-5 cells.

**Methods:**

Cultured RGC-5 cells were exposed to different durations as well as intensities of optical radiation, filtered to exclude wavelengths below 400 nm. Some cells were also subjected to metabolic compromise by depriving them of serum (serum deprivation; SD). Treated cells were assayed for cell viability using the 3,(4,5-dimethylthiazol-2-yl)-2,5-diphenyltetrazolium bromide (MTT) reduction assay, for DNA breakdown by terminal deoxynucleotidyl transferase (TdT)-mediated d-UTP-linked nick end labeling (TUNEL), apoptotic protein activation by immunoblotting, and the production of reactive oxygen species (ROS) with dihydroethidium. A subset of cells was treated with 100 pM rotenone as an alternative means to induce metabolic stress; this was to determine that the influence of light on compromised cells was not specific to serum-deprivation alone.

**Results:**

Exposure to the light for 48 h activated both caspase-3 and Bcl-associated X-protein (Bax) in cultured RGC-5 cells. Furthermore, light (1000 or 4000 lux), SD, and rotenone caused minor but significant decreases in cellular MTT reduction. SD and light also led to cellular DNA breakdown, although only light caused ROS production. Light (48 h) significantly exacerbated the effect of SD on MTT reduction and DNA cleavage. Furthermore, the antioxidant, trolox, significantly blunted the detrimental influence of light on cell viability, increase in TUNEL-positive cells, and the generation of ROS.

**Conclusions:**

Exposure to light was slightly, but significantly, harmful to healthy RGC-5 cells alone, but was much more toxic to those cells that were energetically compromised. Continuous light exposure can therefore detrimentally affect metabolically stressed RGC-5 cells. This may have implications for some ocular retinopathies such as glaucoma

## Introduction

It is accepted that retinal ganglion cells (RGC) die in optic neuropathies, such as glaucoma, as a result of apoptosis [1-3]. Some researchers have hypothesized that this process is initiated by a range of extracellular stimuli, such as glutamate, nitric oxide, or tumour necrosis factor alpha (TNFα), which are released by malfunctioning cells in the region of the optic nerve head [4-6]. The process whereby such mediators can stimulate cell death involves an activation of cell surface receptors and hence is known as the extrinsic pathway of apoptosis [7,8]. However, a second intrinsic pathway also exists [9]. This pathway mediates damage to subcellular constituents, such as mitochondria, and the subsequent intracellular release of factors that also stimulate the cell to undergo apoptotic degradation. The possibility of both types of apoptosis contributing to pathological ganglion cell loss is therefore worthy of consideration.

Ganglion cells are densely laden with mitochondria, which have an asymmetric distribution in the cell. The highest density of these organelles is in the nonmyelinated, intraocular portions of their axons, compared to the myelinated, extraocular portions, which make up the optic nerve [10]. This is conceived to be necessary to provide sufficient energy for nerve transmission in the unmyelinated axons [11] and to maintain a healthy metabolic status for the whole cell. Mitochondria control numerous metabolic reactions within a cell including oxidative processes that generate reactive oxygen species (ROS) as a byproduct [9]. Under physiologic conditions, cells possess several antioxidant defense mechanisms, which prevent such reactive intermediates from becoming toxic [12]. However, it is now well established that sustained or intense cellular insults, such as aging [13] or ischemia [14], can cause mitochondria to produce excess and uncontrolled levels of ROS and that this can cause cell death via the intrinsic apoptotic pathway. We hypothesize that light is an additional factor that can lead to enhanced ROS production in cells.

It has long been known that exposure of biological systems to high energy ultaviolet light can result in the photochemical release of toxic as well as mutagenic end-products [15-20]. Recently, however, studies have shown that components of the visible spectrum can be absorbed by biologic chromophores [21] in cells, such as astrocytes [22] and epithelial cells [23], to cause cellular dysfunction and even death. It is believed that the blue region of the spectrum (approximately 400–500 nm) is particularly likely to induce these reactions, since it has relatively high energy and can penetrate through tissues to cells and their organelles. It can be theorized, therefore, that mitochondria, which are laden with prominent chromophores (e.g., cytochrome C oxidase, flavins, and flavoproteins), can be affected in a detrimental way by components of visible light [24]. This is supported by observations that isolated, light-exposed mitochondria release several ROS, including singlet oxygen, superoxide, and hydroxyl radicals [23]. It is likely that light will have a greater detrimental effect to RGC axons than to more external cellular elements, in part because the axons form the most internal layer and are rich in mitochondria and in part because absorption by this layer will attenuate the delivery of energy to outer layers. At the macula, a further shielding is afforded by carotenoid pigments located in the photoreceptor layer. Therefore, the retinal components that will be most susceptible to any possible light-induced damage are the intraocular ganglion cell axons and in particular, their mitochondria.

The human retina is protected from shorter wavelength radiation by the cornea and lens, which absorb UV light below 400 nm [24]. It is likely that under normal circumstances there is insufficient energy in this light that impinges on the retina to directly cause death of otherwise healthy neurons. It is more likely that light radiation may merely serve as an additional strain for cells already compromised in some way. In glaucoma, for example, it is believed that minor insults residing in the optic nerve head serve to lower the functional status of affected ganglion cell axons [1,25]. We have postulated that such axons may then be susceptible to the detrimental effects of light radiation [26].

The present investigations were designed to test the principle that continuous light exposure can have detrimental effects on cultured RGC-5 cells. Were this to be the case, then it may be possible to hypothesize that ganglion cells could be adversely affected by light exposure, in situ, and this may have some relevance to pathological RGC death in certain ocular diseases.

## Methods

### Materials

The transformed rat RGC line, RGC-5 [27], was a kind gift from Dr. Neeraj Agarwal (Department of Pathology and Anatomy, The University of North Texas Health Science Center, Fort Worth, TX). Cell culture medium and additives were from Invitrogen (Paisley, Scotland) and culture plasticware was from Sarstedt Ltd (Leicester, UK). Terminal deoxynucleotidyl transferase (TdT) and 16-deoxyuridine (d-UTP) were from Promega (Southampton, UK), and dihydroethidium (DHE) was from Boehringer Mannheim (Lewes, UK). Monoclonal mouse anti-actin was from Chemicon (Southampton, UK), rabbit anti-human/rat Bax was from Alexis Corporation (UK distributor, Axxora UK Ltd, Nottingham, UK) and mouse anti-caspase-3 was from Becton Dickenson (Cowley, UK). All other chemicals and reagents were from Sigma Chemical Company (Gillingham, Dorset, UK).

**Figure 1:**
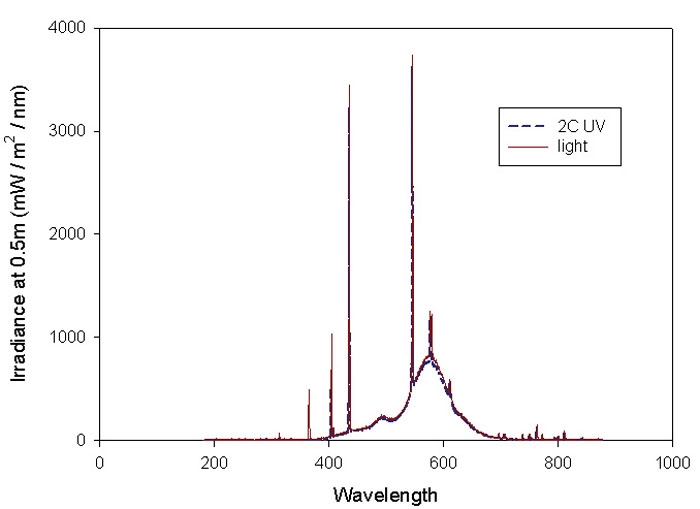
Spectral irradiance profile of the fluorescent light source used. The filtration profile of the Lee 2C ultraviolet (UV) filter is also shown alongside; it is evident that light filtration maintains illuminance of the cells within the visible region of the electromagnetic spectrum.

### Culture of RGC-5 cells

RGC-5 cells have been previously characterized as expressing ganglion cell markers and exhibiting ganglion cell-like behavior in culture [27]. Cells were cultured in Dulbecco’s modified eagle medium (DMEM) containing 5 mM glucose, 100 U/ml penicillin/streptomycin, 2 mM glutamine, 5 mg/ml active geneticin, and 10% (v/v) heat inactivated fetal calf serum in a humidified incubator with 5% CO_2_ at 37 °C. Doubling time of these cells was approximately 20 h under these conditions, and they were generally passaged at a ratio of 1:8. For viability assays, we plated cells onto CellPlus™ Sarstedt Ltd (Leicester, UK) positively charged 96-well plates. To assess reactive oxygen species with DHE and identify apoptotic cells with TdT-mediated d-UTP-linked nick end labeling (TUNEL) analysis, we grew cells on sterilized borosilicate glass coverslips in 24-well plates. For immunoblot analysis, we harvested cells from 75 cm^2^ filter-capped cell culture flasks.

### Treatment of cells and lighting regime

Cells were passaged and used for experiments 24 h later. Serum-deprivation experiments were conducted by washing cells three times with sterilized phosphate buffered saline PBS, pH7.4: 137mM NaCl, 5.4mM KCl, 1.28mM NaH_2_PO_4_, 7mM Na_2_HPO_4_) and then serum-free DMEM (with all other additives present as outlined previously) was added for the appropriate amount of time was added for either 24 or 48 h. In other experiments, the culture medium was changed and then 100 pM rotenone was added into the culture vessel for either 24 or 48 h; the concentration of rotenone was one we had previously determined would depress cellular metabolic function without causing cell death. In some cases, 100 nM-10 μM trolox, an antioxidant, was included once every 24 h in the culture medium.

To create a constant level of illumination, we placed some cells into the same cell culture incubator, with identical ambient conditions to normal cultures except that a standard 8 watt fluorescent strip-light (spectral characteristics shown in Figure 1) was housed 15 cm directly above culture plates to insure that all cultures received the same lighting levels; the light intensity at the plates was 1000 lux, as measured using a light meter. Control plates were also present at the same time, equidistant from the light source, but these were covered with white card hoods, which completely prevented the light from entering. Having control cells in the same conditions except for the covering hoods served to eliminate the possibility that heat increases in the plates were responsible for any observed changes, since any observed differences in cell responses would be independent of temperature effects. The strip-light was completely covered with a filter that excluded optical radiation below 400 nm (UV filter number 2C; Lee Filters, Andover, UK; Figure 1) to confine retinal cell exposure to the visible spectrum. Such a filtered light source still gave rise to several intensity peaks (Figure 1); no attempt was made to ascribe any changes to a particular wavelength of the white light. It was also obvious that the light used did not have identical intensities of light wavelengths as does normal white light; the system was used to ascribe effects to white light that experimentally was *similar* to that impinging on the normal retina in situ. To test the effect of different light intensities, in some investigations, we added an extra light and moved lights closer to the plates, increased lighting level at the plates to 4000 lux. In some initial experiments, medium lacking the photosensitizer, phenol red, was compared with the aforedescribed standard DMEM, and such tests determined that the presence of this compound did not influence the experimental outcome in any situation. Finally, it was determined in preliminary experiments (data not shown) that light did not affect the rate of proliferation of the RGC-5 cells.

### Cell viability determination

The assay used to assess cell viability was the 3,(4,5-dimethylthiazol-2-yl)-2,5-diphenyltetrazolium bromide (MTT) reduction assay modified from that of Mosmann [28]. MTT is reduced to form an insoluble, blue formazan product by accepting electrons from cellular reducing equivalents such as reduced nicotine adenine dinucleotide (NADH) or reduced nicotine adenine dinucleotide phosphate (NADPH), or succinate in living cells [29,30]. It thus acts as a measure of the redox state of the cell, thereby providing quantification of the surviving cells at a given end-point. Furthermore, MTT was found, in control experiments, to be stable in the lighting regimes used in the study.

Briefly, cells were subjected to the appropriate treatments for the required times and then MTT was added to wells at a final concentration of 0.5 mg/ml for a further hour under the same conditions. After this time, medium was removed from the cultures and reduced MTT (blue formazan product) was solubilized by adding 100 μl of dimethyl sulphoxide to each well. After agitation of plates for 15 min, the optical density of the solubilized formazan product in each well was measured using an automatic microplate reader (Titertek Plus MS212; ICN Flow, Thame, UK) with a 570 nm test wavelength and a 690 nm reference wavelength. Medium plus or minus fetal calf serum controls were also performed to determine that the presence of this additive did not influence absorbance readings directly.

**Figure 2:**
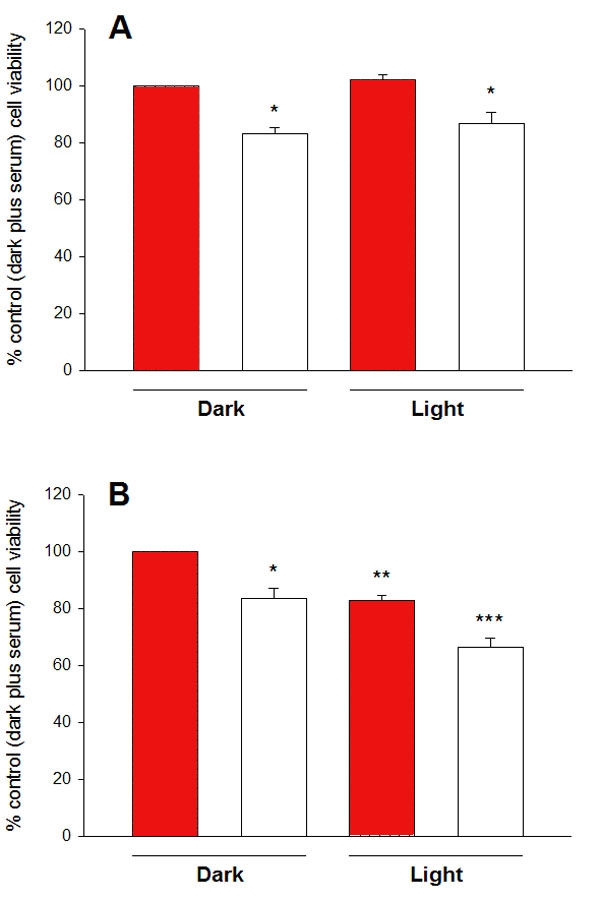
The effect of serum deprivation and filtered light on viability of cultured RGC-5 cells. Cells were incubated in normal culture conditions in the presence (red bars) or absence (empty bars) of 10% (v/v) serum and in the presence or absence of light of intensity 1000 lux, as shown for **A** 24 h or **B** 48 h. After treatment, the MTT reduction assay was used to determine the level of cell survival, compared with controls. Serum deprivation caused a small but significant reduction in culture viability, as compared with cells incubated with serum in the dark. This effect was independent of the time of incubation (24 or 48 h). Furthermore, filtered light itself caused a significant reduction in cell viability, but only after 48 h. The toxic effects of both the light treatment and the serum deprivation were additive after 48 h. Statistical analysis using the one-way ANOVA followed by a post-hoc Bonferroni test and comparing with controls incubated in the dark for the same length of time in the presence of serum (n=10), showed significance levels denoted as follows: *p <0.05, ** *p* <0.01, ***p <0.001.

### Assessment of DNA breakdown with the d-UTP-linked nick end labeling procedure

The TUNEL procedure was performed by fixing treated cells on coverslips in 4% paraformaldehyde for 20 min, washing them in PBS containing 0.1% triton X-100 for 10 min, and immersing them in PBS. The labeling procedure was performed exactly as described previously [31]. Briefly, cells were washed in Tris buffer (10 mM Tris-HCl, pH 8.0) and then exposed to 1% (v/v) H_2_O_2_ for 5 min before being incubated in TdT buffer (30 mM Tris-HCl, pH 7.2, plus 140 mM sodium cacodylate and 1 mM cobalt chloride) containing 0.25 units/μl of TdT and 40 μM biotin-16-dUTP, for 60 min at 37^o^C for the transferase reaction. Reaction was stopped by incubation in sodium citrate buffer (300 mM NaCl, 30 mM sodium citrate) before blocking with 2% (w/v) bovine serum albumin in PBS. Positive labelling was developed with avidin-biotin-peroxidase complex solution using 3’3’diaminobenzidine (0.5 mg/ml) and 0.1% (v/v) H_2_O_2_ as substrates. The labeled RGC-5 cells on coverslips were washed in PBS, mounted on glass slides, and visualized with a Zeiss light microscope. Cells on coverslips were lightly counterstained with a 3 s immersion in Gill’s hematoxylin (diluted to 33%, v/v, in water) to visualize unlabeled nuclei. Some cells were treated after fixation but before TUNEL staining with DNase I (0.1 mg/ml) for 15 min at 37 °C to determine that the labeling procedure correctly identified DNA breakdown in nuclei (see [31]).

To quantify the numbers of TUNEL-labeled nuclei, we obtained counts and averaged them from five different randomly selected areas of a given coverslip, using an eyepiece graticule grid that represented an area of 400 μm x 400 μm. Thus, to convert values to cells/mm^2^, each averaged value was multiplied by 6.25 (ie. 2.5×2.5). Ten coverslips were analyzed for each treatment and values statistically compared for differences.

### Assessment of reactive oxygen species production

Cells were assessed for the production of ROS using the dye, DHE [32]. DHE is a nonfluorescent, reduced form of ethidium that can passively cross plasma membranes of live cells. When DHE is oxidized to ethidium by ROS, it can bind to DNA and yield red fluorescence (excitation 475 nm/emission 610 nm). To visualize ROS in this manner, 30 min before the end of the appropriate analyses, we added 5 μM DHE to the medium of cells under investigation. After incubation, cells were fixed in 4% paraformaldehyde for 15 min, washed in PBS and visualized by light microscopy, using a Zeiss microscope with epifluorescence optics. Investigations were performed on four separate cultures, with replicates of two to six coverslips per culture analyzed.

### Analysis of levels of pro-apoptotic proteins by electrophoresis/immunoblot

RGC-5 cells were harvested by scraping into PBS and then cell pellets sonicated in freshly prepared 20 mM Tris/HCl buffer (pH 7.4) containing 2 mM EDTA, 0.5 mM ethylene-glycol-tetracetic acid, 1 mM dithiothreitol, and the protease inhibitors: 0.1mM phenylmethyl-sulphonyl fluoride (PMSF), 50 μg/ml leupeptin, 50 μg/ml aprotinin, and50 μg/ml pepstatin A. An equal volume of sample buffer (62.5 mM Tris/HCl, pH 7.4, plus 4% sodium dodecyl sulfate, 10% glycerol, 10% β-mercaptoethanol, and 0.002% bromophenol blue) was added, and samples were boiled for 3 min. An aliquot was taken at this stage for determination of protein content. Electrophoresis of samples was performed using 10% polyacrylamide gels containing 0.1% sodium dodecyl sulfate [33]. Samples were then transferred onto nitrocellulose overnight [34]. Nitrocellulose blots were probed with either monoclonal anti-caspase-3 (clone 46; 1:1000; recognizes cleaved form of caspase-3), or rabbit polyclonal anti-Bax (1:1000) for 3 h at room temperature and appropriate secondary antibodies conjugated to horseradish peroxidase subsequently used. Nitrocellulose blots were developed with a 0.016% solution of 3-amino-9-ethylcarbazole in 50mM sodium acetate (pH 5) containing 0.05% Tween-20 and 0.03% H_2_O_2_. Proteins were analyzed by densitometry using the LabWorks Image Acquisition and Analysis Software package (UVP Ltd, Cambridge, UK). The presence of actin (1:2000) was assessed in all cell extracts for reference.

### Statistical analyses

All data are presented as mean ± SEM for the indicated number of experiments. To determine significant differences between groups of data after experimental treatments, we analyzed values by one-way ANOVA followed by a posthoc Bonferroni test using SPSS version 12.0. A p-value of less than 0.05 was considered significant.

## Results

### Cell viability

Figure 2 shows the influence of light with an intensity of 1000 lux on the viability of cultured RGC-5 cells. The initial 24 h exposure to light had no significant effect on the ability of RGC-5 cells to reduce MTT (Figure 2A). After a further 24 h, however, there was a reduction to 82.8 ± 1.8% compared with the value for cells in the dark (Figure 2B). Light with an intensity of 4000 lux did not significantly alter this effect (Table 1).

**Table 1:** Effect of different light intensities on serum-deprivation or rotenone-induced cell viability decrease in cultured RGC-5 cells.

Treatment	% viability (compared to dark plus serum control)	n
Dark + serum	100	10
Dark – serum	83.48±56*	10
1000 lux light + serum	82.87±1.78**	10
1000 lux light – serum	66.59±3.03***	10
4000 lux light + serum	77.81±4.19**	4
4000 lux light – serum	†53.66±4.51***	4
100 pM rotenone + dark	88.48±1.33*	8
100 pM rotenone + 1000 lux light	68.90±6.12**	8
+ 10 mM trolox	95.33±4.64‡	4
100 pM rotenone + 4000 lux light	56.22±4.10 **	4

Culture viability was determined by assaying cells for their ability to reduce the dye, 3,(4,5-dimethylthiazol-2-yl)-2,5-diphenyltetrazolium bromide (MTT) to an insoluble blue formazan product after treatments for 48 h. The various treatments were performed as defined in the table; rotenone was applied in the presence of serum. The data clearly show that the increased light exposure levels (4000 lux) had a greater effect on exacerbation of cell damage caused by either serum-deprivation or rotenone than the lower light level (1000 lux). Data are shown as mean ± SEM and related as a percentage of dark + serum control and statistical significance denoted by *p <0.05, ** *p* <0.01, or ***p <0.001, when a one-way ANOVA followed by a post-hoc Bonferroni test was performed, and when when comparing with controls incubated in the dark for 48 h in the presence of 10% (v/v) serum. Further significance is denoted as follows: †*p*<0.05, when comparing indicated samples with cells exposed to 1000 lux light in the absence of serum; ‡*p*<0.05, when comparing indicated samples with cells exposed to 100 pM rotenone and 1000 lux light in the presence of serum.

Depriving cells of serum had the effect of reducing measured viability to 83.1±2.1% of control levels at 24 h (Figure 2A) and 83.5±3.6% at 48 h (Figure 2B). Interestingly, light (1000 lux) significantly exacerbated the effect of serum deprivation on cell viability, but only when measured after 48 h (reduction of viability to 66.6±3% of control; p<0.001). This exacerbatory effect was further enhanced by light with an intensity of 4000 lux as compared to 1000 lux (Table 1).

The effect of a 48 h incubation with 100 pM rotenone was of a similar magnitude to that of serum deprivation (88.5±1.3%; Table 1), and this, too, was exacerbated in the presence of light (1000 lux, 68.90±6.12% of control; Table 1). The effect of the greater light intensity (4000 lux; Table 1) was to cause a decrease in the amount of MTT reduction by cells treated with 100 pM rotenone compared with the light of 1000 lux (56.22±4; 10% of control), but this decrease was not statistically significant.

Finally, subconfluent cells were analyzed in a similar way to determine whether light was able to increase the rate of proliferation or turnover of RGC-5 cells, and this was not the case (data not shown). Thus, the mitochondrial respiratory activity measured in the RGC-5 cells incubated in the dark compared with the light was relative and not influenced by proliferation rates of the cells.

### Assessment of DNA breakdown with the d-UTP-linked nick end labeling methodology

**Figure 3:**
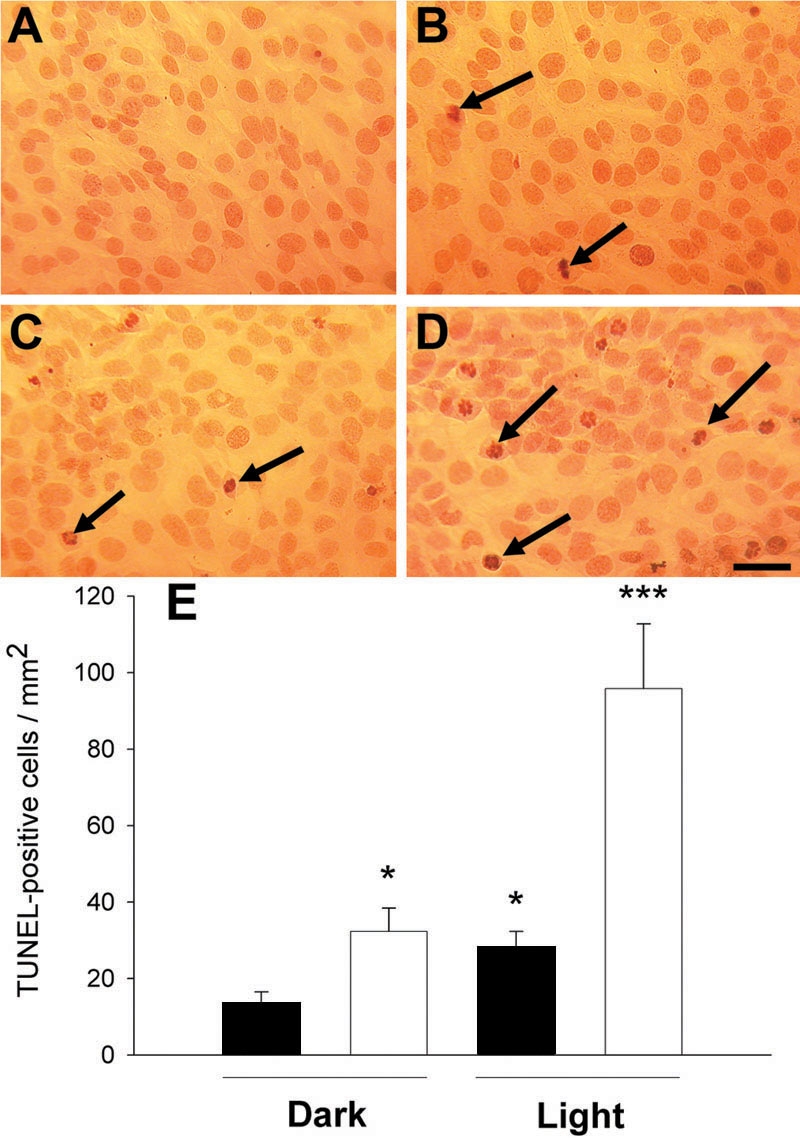
The appearance of DNA damage after light exposure. These experiments were conducted in normal culture medium (A, C) and medium deprived of serum (B, D). Cultures were exposed to light, 1000 lux, for 48 h (C, D) or maintained in the dark (A, B) and processed for the localization of breakdown of DNA (TUNEL). It can be seen that light enhanced the numbers of TUNEL-positive cells (arrows) and this was greatest in serum-free conditions. The scale bar represents a distance of 20 μm. Quantification of several experiments for each condition shown in A-D is shown in E (black bars represent cells incubated with serum, while empty bars indicate cells incubated without serum). Statistical analysis using the one-way ANOVA followed by a post-hoc Bonferroni test and comparing with controls incubated in the dark for the same length of time in the presence of serum (n=10), showed significance levels denoted as follows: *p <0.05, ***p <0.001.

Both filtered light at 1000 lux (Figure 3C, E) and serum deprivation (Figure 3B, E) caused a significant increase in the amount of cultured RGC-5 cell nuclei that labeled positively for TUNEL after 48 h. When both light treatment and serum deprivation were concurrent for 48 h, the effect was to significantly enhance the influence of either treatment alone (Figure 3D, E).

### Visualization of reactive oxygen species production by dihydroethidium labeling

**Figure 4:**
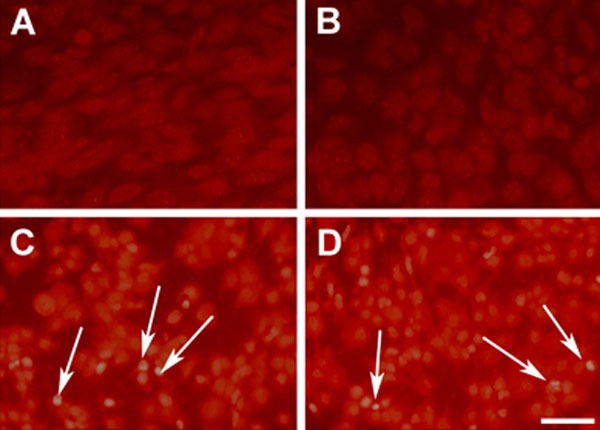
Dihydroethidium labeling of RGC-5 cells. Dihydroethidium labeling of RGC-5 cells showed that only filtered light (1000 lux; C) and not serum deprivation (B) caused the detectable production of reactive oxygen species (red fluorescence, as marked by the arrows), as compared with control cells incubated in the dark in the presence of 10% (v/v) serum (A), after 48 h. This effect did not appear to be obviously enhanced for cells incubated in serum free medium and exposed to light (D). The scale bar represents a distance of 20 μm.

To evaluate the possible involvement of ROS in the cytotoxic effects of light, we treated cells with DHE and examined them under epifluorescence microscopy as described in Methods. Control cells incubated in the dark for 48 h, either with serum (Figure 4A) or without (Figure 4B) exhibited a very low level of fluorescence. However, after exposure to light of intensity 1000 lux for 48 h, cells emitted a high intensity red fluorescence. There was no obvious difference in cells incubated in the absence (Figure 4D) or presence (Figure 4C) of serum. These data confirmed that light and not serum deprivation lead to a significant level of ROS production in cultured RGC-5 cells.

### Pro-apoptotic protein expression

**Figure 5:**
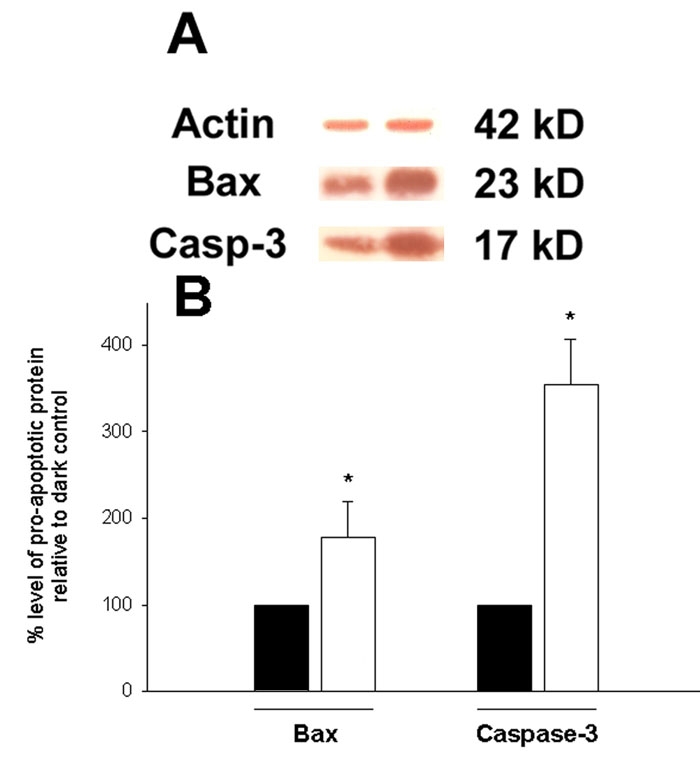
Changes in cellular pro-apoptotic proteins. RGC-5 cells incubated in filtered light for 48 h showed an increased production of pro-apoptotic Bax and cleaved (17 kD) caspase-3 proteins compared with cells grown in the dark (A). Quantification by densitometric analysis of protein levels showed the effect to be significant in both cases (*p<0.05, by one-way ANOVA followed by a post-hoc Bonferroni test; n=4). All extracts were prepared, in these experiments, from cells incubated in the presence of serum (black bars represent cells incubated in the dark and unfilled bars in the light).

As shown in Figure 5, RGC-5 cells (in the presence of serum) exposed to light (1000 lux) for 48 h exhibited significant increases in the levels of the cleaved, active 17 kD-form of caspase-3 and of Bax protein (23 kD), relative to the level of actin present in each sample. Both of these protein forms have been associated with apoptotic death of neurons [35,36].

### Counteraction of light effects with trolox

**Figure 6:**
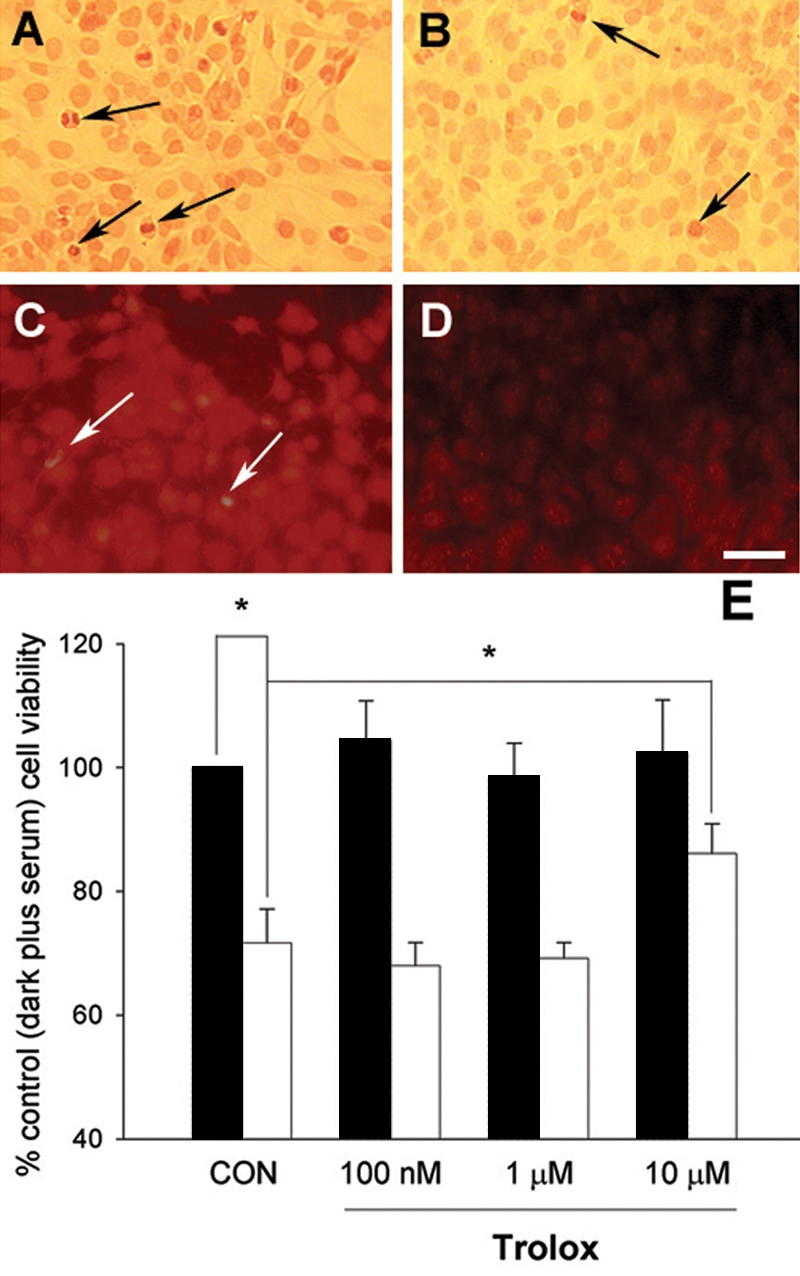
The counteracting effect of trolox. Cell viability assays with 3,(4,5-dimethylthiazol-2-yl)-2,5-diphenyltetrazolium bromide (MTT) showed that 10 µM trolox significantly blunted the detrimental effect of light (1000 lux, 48 h) in serum-free medium (white, unfilled bars) compared with cells incubated in normal medium in the dark (black bars; Figure 6E). Moreover, analysis of DNA breakdown by TUNEL (Figure 6A and 6B) or ROS formation (Figure 6C and 6D) showed that the detrimental effect of light (Figure 6A and 6C) was counteracted by inclusion in the medium of 10 µM trolox (B, D). Black arrows indicate TUNEL-positive cells. White arrows mark red fluorescence in cells staining strongly for ROS (DHE labeling). The scale bar represents a distance of 20 μm. Statistical significance, as indicated (*p <0.05), was determined by one-way ANOVA followed by a post-hoc Bonferroni test which either compared cells in the light (without serum) to controls, or compared cells in the light (without serum) with or without 10 μM trolox, as indicated in the figure. Note that trolox had no significant effect on the viability measurement of cells incubated in the dark, in medium containing serum.

Further support for the involvement of ROS in the cytotoxic effect of light on RGC-5 cells is shown in Figure 6. These studies examined the influence of the antioxidant trolox on the light-induced stimulation (1000 lux in absence of serum for 48 h) of TUNEL positive cells (Figure 6A and 6B), ROS (Figure 6C and 6D) and viability as assessed by the MTT assay (Figure 6E). Figure 6E shows that 10 µM trolox (86.08 ± 4.84% of control value) significantly blunted the detrimental influence of light (71.65±5.50 of control value) on cell viability, while having no effect on cells incubated in the dark in the presence of serum. This concentration of trolox also clearly reduced the numbers of TUNEL positive cells (Figure 6A and 6B) and generation of ROS (Figure 6C and 6D). Lower concentrations of trolox (1 μM, 69.13±2.60% of control; 100 nM, 67.97±3.73% of control) had no significant effect on reversing the detrimental effects of light on RGC-5 cells (Figure 6E). Trolox (10 μm) was also able to counteract the decrease in MTT reduction by the cells caused by 100 pM rotenone in the presence of light (1000 lux; 95.33±4.64% of untreated control value; Table 1).

## Discussion

To test our recently proposed hypothesis that light entering the globe might act as an additional risk factor to compromised RGC [26], we analyzed cultured cells, enabling close control of the duration and intensity of light exposure. One obvious difficulty in working with isolated ganglion cells is obtaining sufficient numbers of such cells to generate meaningful data. We therefore used the recently described rat RGC-5 cell line [27]. These cells are derived from and possess characteristics of normal RGC and form a homogeneous population in culture. For our lighting paradigm, we used a light filter that absorbs wavelengths below 400 nm to simulate the processes that may occur to ganglion cells in the intact eye. In addition, we placed cultured cells under independent stress by either removing serum, and hence nutrients and growth factors, or by treating with a low, sublethal concentration of the mitochondrial complex I inhibitor, rotenone.

In the present study we demonstrated two effects: 1) exposure to optical radiation in the visible spectrum can reduce the viability of cultured RGC-5 cells; and 2) the same light-exposure paradigm enhanced the damaging effects to RGC-5 cells of being deprived of serum. These data fit well with our recently proposed theory, suggesting that physiologic exposure of the retina to light may provide an additional stress for ganglion cells already compromised energetically by other factors [26]. We have hypothesized, for example, that ganglion cells may be compromised in diseases like glaucoma, where RGC axons may suffer ischemic stress at the optic nerve head, or in inherited disorders affecting mitochondrial function, such as Leber Hereditary Optic Neuropathy (LHON) and autosomal dominant optic atrophy (ADOA type 1). In these latter disorders there is abundant evidence of defective ATP production [37,38].

In the present study, to simulate retinal exposure in situ, we exposed RGC-5 cells to white light, using a filter to remove wavelengths below 400 nm but including several intensity peaks (Figure 1). The white light source that was used was not identical to normal light, but showed a range of intensity peaks that were spread over a similar range to that seen in the spectral characteristics of white light. It is generally accepted that the shorter wavelengths of light pose the greatest potential hazard to biologic systems because they contain the most energy [15]. Indeed, blue light, which is the component of visible light reaching the retina with the lowest wavelength and hence greatest energy [39], has been shown to induce retinal damage, particularly to the retinal pigmented epithelium [23,32,40-43] and to photoreceptors [44-47], by a process involving the production of reactive oxygen intermediates. It is of interest to note in the present study that there was an intensity peak in the filtered light impinging on the cells which corresponded to the blue part of the electromagnetic spectrum, but no attempt was made to ascribe any of the effects to this component of white light; the suggestion that the blue component of light may be the destructive one may, perhaps, be inferred from other studies [23,32,40-47]. The process by which light can stimulate production of reactive oxygen intermediates in cells involves its interaction with a photosensitizer (chromophore) which subsequently interacts with neighboring molecules to form radicals [24]. The retina contains a large number of photosensitizers, which readily absorb visible light, including retinoids, melanin, and the lipofuscin-chromophore A2E, as well as the mitochondrial flavins, flavoproteins, and cytochromes [12,21,48-50]. Light would be expected to have a greater detrimental effect on inner retinal cells such as ganglion cells because outer retinal cells express the macular carotenoid pigments, which are known to partially protect against (blue) light-induced damage [51,52]. Furthermore, a novel class of ganglion cells has recently been described--the intrinsically photosensitive retinal ganglion cells--and these express the light-sensitive photopigment, melanopsin [53,54]. However, in the human retina, these cells represent a small fraction of the total number of RGCs [55]. It is not yet known whether such pigments represent a relevant target for any potentially toxic effects of light.

We have previously demonstrated that when exposed to the lighting regime used in the present study, isolated mitochondria showed a general depression of function [26]. Furthermore, isolated mitochondria exposed to light have been demonstrated to generate singlet oxygen, superoxide anions, and hydroxyl radicals [23]. In this case, mitochondrial DNA damage was also noted and the use of different classes of antioxidants proved that this damage was predominantly mediated by superoxide anions [23]. In the present study, DHE labeling was employed to demonstrate the production of reactive oxygen intermediates in RGC-5 cells exposed to light. This technique has been widely used to identify the production of ROS in isolated mitochondria [56], RPE cells [32], and in neurones [57], and it preferentially detects superoxide radicals [58]. That DHE labeling was shown here to be intensified in light-exposed RGC-5 cells suggests that the detrimental effects of light were mediated, at least in part, through a production of superoxide.

The idea that light can eventually cause apoptosis is a logical one, since the stimulation of ROS production in cells is known to play an intricate role in the activation of intracellular death pathways [9]. It is now believed that ROS are central to the intrinsic system of apoptosis [13]. Indeed, previous work has shown that RGC-5 cells themselves die by a process that is suggestive of apoptosis when exposed to glutathione depletion, t-butyl hydroperoxide, or hydrogen peroxide, all of which will overcome cellular antioxidant defense mechanisms [59,60]. In the present study, light alone does stimulate the apparent apoptotic death of some RGC-5 cells as shown by the increased number of TUNEL-labeled nuclei and by the appearance of active pro-apoptotic protein markers after 48 h. These results again suggested that light stimulated production of reactive oxygen intermediates in the cells to induce apoptosis. Only a small percentage of cells actually exhibited DNA breakdown after 48 h exposure to light alone, although the detection of active caspase-3 and Bax at this stage did suggest that more cells were being directed toward apoptosis. The increased loss of culture viability after being exposed to an illumination of 4000 lux also suggested that light of a greater intensity or of a longer duration may have stimulated more cells to die, without them being subjected to any other independent stress. However, these observations were not investigated further, since the cells themselves started to detach from their substratum after 72 h, probably due to their being fully confluent. The experiments performed in the presence of the water-soluble vitamin E analog and antioxidant, trolox, provided further evidence that light stimulated ROS production in RGC-5 cells; trolox was able to prevent the increased fluorescence seen by labeling with DHE, and also significantly abrogated the decrease in cell viability and appearance of TUNEL-positive nuclei induced by light exposure. The activation of caspase-3 and Bax in this study fits with the notion that light stimulated induction of the intrinsic apoptotic pathway. The extrinsic pathway is known to be regulated by extracellular stimuli that signal the induction of apoptosis by activating caspase-8 and caspase-3, in sequence [7]. The intrinsic pathway, however, involves mitochondrial events such as Bax activation, permeability transition, and cytochrome C release [61]. Caspase-3 is also activated in this process, but this time it may be activated by caspase-9. Therefore, although caspase-3 activation is not indicative of the specific apoptotic pathway involved, since it acts as a downstream effector for different apoptotic stimulatory events, the activation of Bax does implicate the intrinsic pathway [61].

More pertinent was the observation that light was able to enhance the small decrease in viability detected after either serum withdrawal or rotenone treatment. Cells were deprived of serum as a means to remove their external supply of growth factors, although this insult can also result in mitochondrial complex I inhibition [62] and a minor elevation in intracellular levels of ROS [63]. A previous study has used this procedure to describe, in detail, how removal of trophic support can kill ganglion cells by apoptosis, as has been suggested to be the case in glaucoma after cessation of retrograde axoplasmic flow in the optic nerve head [64]. The authors described how RGC-5 cells died following serum withdrawal, and that apoptotic gene and protein expression was evident after two days. They noted, however, that there was a 50% loss of cells after only two days of serum withdrawal, which was significantly greater than that seen in the present study. They determined this by using the neutral red viability assay, however, and it is known that different means of assaying cell viability utilize different end-points [30]. Rotenone is a mitochondrial complex I inhibitor that also stimulates the production of ROS above physiologic levels [65]. In the present study, rotenone was used at a concentration that affected mitochondrial function without causing large numbers of RGC-5 cells to die. This was evidenced by the small, but significant decrease in viability of cells after treatment with this compound alone. It was therefore demonstrated that either serum withdrawal or rotenone application are able to lower the viability of RGC-5 cells, without leading to widespread cell death. The finding that in both cases light, in an intensity-dependent manner, was able to significantly enhance measured cytotoxicity, provided important evidence for our hypothesis that light exposure can act as a further stress to compromised ganglion cells. Both serum deprivation and rotenone can compromise cellular function by preventing full mitochondrial function and stimulating the basal production of ROS, thereby saturating or inactivating cellular antioxidant defense mechanisms. Thus, in both experimental situations, it is likely that the enhanced stimulation of reactive oxygen intermediates by light added an excess strain to cells that could no longer provide an adequate defense and this resulted in the death of a larger number of cells. This was further evidenced by the ability of trolox to protect, in both serum-deprived and rotenone-treated cells. It is hypothetically possible that such a process could take place in ganglion cells in situ during optic neuropathies such as ADOA, LHON, or glaucoma [66].

In summary, the present data clearly demonstrate that while providing only a minor insult to healthy cells, light exposure causes much more widespread death of RGC-5 cells that have been energetically compromised. It must be noted that the light levels used in the present study as an experimental tool are relatively excessive to that which would impinge upon the retina in all everyday situations. If light exposure is confirmed as a risk factor in optic neuropathies such as glaucoma, LHON, or ADOA, however, then the use of devices or behavioral responses to minimize retinal exposure at the relevant wavelengths, has important therapeutic potential [67,68].
